# Reviewers in 2016

**DOI:** 10.1098/rsob.170092

**Published:** 2017-05-10

**Authors:** David M. Glover

**Affiliations:** Editor-in-Chief

The Editors of *Open Biology* would like to thank all reviewers who have worked on articles published in the journal in 2016. We appreciate that most reviewers value recognition of their efforts by the journal and through services such as *Publons*, which is partnered with *Open Biology.*

To provide an opportunity for recognition, we list the names of all reviewers (who opted for inclusion). This article is made permanent and citeable by its digital object identifier (DOI). We encourage you to quote your contribution in your applications for tenure and grants or other forms of research assessment. Where you have provided it, we have included your ORCID so your contribution can be unambiguously assigned to you.

Lun ATL
Hachani A
Diwan A
Blanchard A
Quintana A
Hergovich A
Miserez A
Maloyan A
Carmena A
Musacchio A
Preiss A
von Philipsborn AC
Lambeir A-M
Boudaoud A
Queliconi BB
Coutard B
Larsen C
Norden C
Prahalathan C
Faherty CS
Cooper CDO
http://orcid.org/0000-0002-9197-8041
Scheffers D-J
Heymann D
Young R
Caspari T
Tromer E
http://orcid.org/0000-0003-3540-7727
Lou E
Huber EM
Zietkiewicz E
Cernilogar FM
Uhlmann F
Macintyre G
Polese G
van Wezel GP
Lillywhite HB
http://orcid.org/0000-0002-6410-4832
Huang H-H
Djagaeva I
Davie JR
Tagne J-B
Chen J
Yang J
Zhang J
http://orcid.org//0000-0001-8683-509X
Nilvebrant J
Atkins JF
Higgins J
Tatzelt J
Jimenez JI
Rood JI
Welburn J
Motohashi K
http://orcid.org/0000-0002-8414-2836
Hermo L
Koziol MJ
Peres MA
Giansanti MG
http://orcid.org/0000-0002-6753-7262
Peifer M
Waldor M
Yun MH
Shao M
Laurent MR
Sendtner M
Härkönen P
Diederichs S
Kaufman J
http://orcid.org/0000-0002-7216-8422
Gaur R
Das RM
Strugnell RA
Haltiwanger RS
Khochbin S
Wu S
Yeh S-D
Cragg SM
Araújo SJ
Rocha S
Scholpp S
Sweet SM
Evans SE
Mitchell TJ
Tanaka T
Bollag WB
Yang X
http://orcid.org/0000-0002-2537-5752
Yoshida K

